# Establishment of an antibody specific for cancer-associated haptoglobin: a possible implication of clinical investigation

**DOI:** 10.18632/oncotarget.24332

**Published:** 2018-01-29

**Authors:** Kimihiro Nishino, Sayaka Koda, Naoya Kataoka, Shinji Takamatsu, Miyako Nakano, Shun Ikeda, Yuka Kamamatsu, Koichi Morishita, Kenta Moriwaki, Hidetoshi Eguchi, Eiko Yamamoto, Fumitaka Kikkawa, Yasuhiko Tomita, Yoshihiro Kamada, Eiji Miyoshi

**Affiliations:** ^1^ Department of Molecular Biochemistry and Clinical Investigation, Osaka University Graduate School of Medicine, Suita, Osaka, Japan; ^2^ Department of Obstetrics and Gynecology, Nagoya University Graduate School of Medicine, Nagoya, Aichi, Japan; ^3^ Graduate School of Advanced Sciences of Matter, Hiroshima University, Higashihiroshima, Hiroshima, Japan; ^4^ Department of Gastroenterological Surgery, Osaka University Graduate School of Medicine, Suita, Osaka, Japan; ^5^ Department of Healthcare Administration, Nagoya University Graduate School of Medicine, Nagoya, Aichi, Japan; ^6^ Department of Pathology, International University of Health and Welfare, Narita, Chiba, Japan

**Keywords:** fucosylation, glycosylation, haptoglobin, glycan antibody, cancer biomarker

## Abstract

We previously found that the serum level of fucosylated haptoglobin (Fuc-Hpt) was significantly increased in pancreatic cancer patients. To delineate the mechanism underlying this increase and develop a simple detection method, we set out to generate a monoclonal antibody (mAb) specific for Fuc-Hpt. After multiple screenings by enzyme-linked immunosorbent assay (ELISA), a 10-7G mAb was identified as being highly specific for Fuc-Hpt generated in a cell line as well as for Hpt derived from a pancreatic cancer patient. As a result from affinity chromatography with 10-7G mAb, followed by lectin blot and mass spectrometry analyses, it was found that 10-7G mAb predominantly recognized both Fuc-Hpt and prohaptoglobin (proHpt), which was also fucosylated. In immunohistochemical analyses, hepatocytes surrounding metastasized cancer cells were stained by the 10-7G mAb, but neither the original cancer cells themselves nor normal hepatocytes exhibited positive staining, suggesting that metastasized cancer cells promote Fuc-Hpt production in adjacent hepatocytes. Serum level of Fuc-Hpt determined with newly developed ELISA system using the 10-7G mAb, was increased in patients of pancreatic and colorectal cancer. Interestingly, dramatic increases in Fuc-Hpt levels were observed at the stage IV of colorectal cancer. These results indicate that the 10-7G mAb developed is a promising antibody which recognizes Fuc-Hpt and could be a useful diagnostic tool for detecting liver metastasis of cancer.

## INTRODUCTION

It is a well-known fact that the oligosaccharide structure on glycoproteins is altered in several pathological conditions, including cancer [1]. Fucosylation is the attachment of fucose on glycans via α1-4, and α1-6 linkages. Fucosylation is one of the most important types of glycosylation associated with cancer and inflammation [2]. We previously reported increased levels of fucosylated haptoglobin (Fuc-Hpt) in the sera of patients with pancreatic cancer, based on lectin blot analysis using *Aleuria aurantia lectin* (AAL), which recognizes all types of fucosylation [3]. To facilitate the quantitative measurement of serum Fuc-Hpt levels, we established a lectin-antibody enzyme-linked immunosorbent assay (ELISA) for Fuc-Hpt [4] and evaluated the lectin-antibody ELISA system under various conditions [5]. Based on the results of receiver operating characteristic (ROC) analysis, we demonstrated that the sensitivity and specificity for serum Fuc-Hpt levels for pancreatic cancer diagnosis were 85.1% and 82.3%, respectively (area under curve [AUC], 0.91). The diagnostic efficacy of this method for pancreatic cancer was almost the same as that of carbohydrate antigen 19-9 (CA19-9) which is commonly used for the diagnosis of pancreatic cancer. Serum Fuc-Hpt levels increased with the progression of pancreatic cancer through its clinical stages and were especially high at stage IV [4]. In general, many cancer biomarkers such as α-fetoprotein, carcinoembryonic antigen (CEA) and CA19-9 are produced by cancer cells themselves. However, it remains unknown whether or not Fuc-Hpt is produced by pancreatic cancer tissue and/or the liver. Our previous studies demonstrated high expression of Fuc-Hpt in the human pancreatic cancer cell line PSN-1, but not in other pancreatic cancer cell lines [3].

In contrast, serum Fuc-Hpt levels are observed to increase in other cancers and liver diseases [6–8]. A combination assay for Fuc-Hpt and CEA is a prognostic cancer biomarker for colorectal carcinoma [9]. Serum Fuc-Hpt levels increased with liver disease progression from normal volunteers, to chronic hepatitis patients, to liver cirrhosis patients, and could be a potential predictive biomarker for hepatocellular carcinoma [10]. We also found that serum Fuc-Hpt is a useful biomarker for non-alcoholic steatohepatitis (NASH) especially for the prediction of ballooning hepatocytes (characteristic pathological observation of NASH) [11, 12]. When we used *Pholiota squarrosa* lectin (PhoSL), which recognizes core fucose (α1-6 fucose) more specifically, instead of AAL, in the lectin-antibody ELISA, serum levels of each lectin-reactive Hpt were not the same [13]. These observations suggest that Fuc-Hpt is produced by various kinds of cells, and that the fucosylation linkage differs in a disease-specific manner. To identify a productive source of Fuc-Hpt and to elucidate a mechanism underlying the increase of Fuc-Hpt, a specific antibody for Fuc-Hpt is required that can be used in immunohistochemical study and/or ELISA.

In the present study, we have developed novel antibodies specific for Fuc-Hpt with our unique strategy, using HCT116 cells in which fucosylation is completely absent due to a mutation in the GDP-mannose-4, 6-dehydratase (*GMDS*) gene [14] and can be restored with L-fucose treatment. The characterization and possible clinical usefulness of one of the established antibodies, named the 10-7G mAb, were investigated. Furthermore, the availability of our preliminary ELISA system using novel Fuc-Hpt antibodies was investigated in terms of a diagnostic biomarker for pancreatic cancer and colorectal cancer.

## RESULTS

### Establishment of specific antibodies against fucosylated haptoglobin

**Figure 1:**
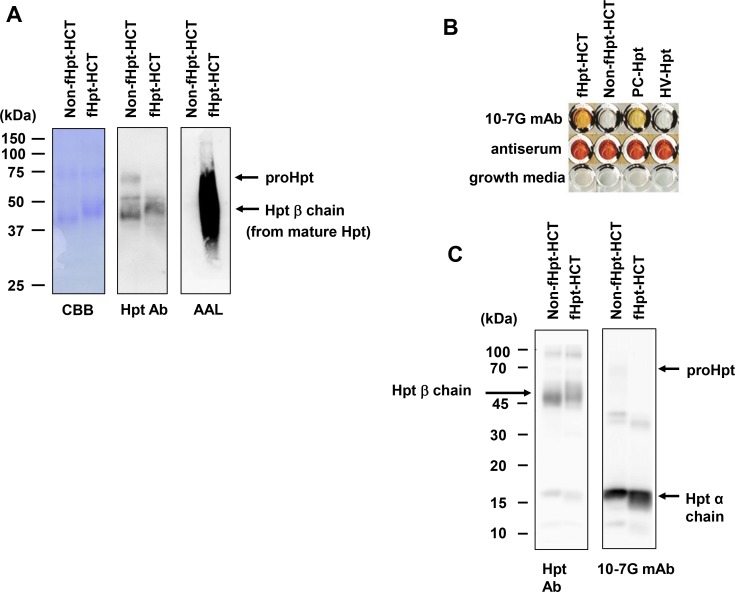
Establishment of specific antibodies against fucosylated haptoglobin (**A**) Western blot and AAL lectin blot analyses on Non-fHpt-HCT and fHpt-HCT purified from conditioned media from Hpt-transfected HCT116 cells. (**B**) The 10-7G mAb was reacted to fHpt-HCT and PC-Hpt (Hpt derived from a pancreatic cancer patient), but not to Non-fHpt-HCT, and HV-Hpt (Hpt derived from a healthy volunteer) in ELISA. (**C**) Western blot analyses of fHpt-HCT, Non-fHpt-HCT with the polyclonal Hpt Ab and the 10-7G mAb.

We purified approximately 1 mg of Hpt from conditioned media from Hpt-transfected HCT116 cells with or without L-fucose supplementation (fHpt-HCT or Non-fHpt-HCT). As shown by the CBB staining in Figure 1A, each purified Hpt contained a high amount of the 40-kDa Hpt β chain from mature Hpt and a low amount of 70-kDa prohaptoglobin (proHpt) (left panel in Figure 1A). Western blot analysis using a commercial polyclonal antibody (Hpt Ab, Dako) showed that the molecular weight of the Hpt β chain in fHpt-HCT was slightly higher than that in Non-fHpt-HCT (center panel in Figure 1A). AAL blot analysis confirmed complete deficiency and restoration of fucosylation in Non-fHpt-HCT and fHpt-HCT, respectively (right panel in Figure 1A). Next, we immunized Balb/c mice at 6 weeks of age with purified fHpt-HCT, extracted splenocytes four weeks after immunization, and generated hybridomas with mouse myeloma cells according to a standard procedure. Conditioned media from growing hybridomas were screened with ELISA as follows. In the first screening, we selected hybridomas containing antibodies that showed positive reaction to fHpt-HCT and negative reaction to Non-fHpt-HCT. Positive clones from the first screening were applied to the second screening, in which clones producing antibodies that showed positive reaction to Hpt derived from a pancreatic cancer patient (PC-Hpt) and negative reaction to that derived from a healthy volunteer (HV-Hpt) were selected. A few hybridoma clones produced antibodies that were highly specific for fHpt-HCT and PC-Hpt. After limiting dilution of the hybridomas, seven clones producing monoclonal antibodies specific for both fHpt-HCT and PC-Hpt were established (Figure 1B). Among these antibodies, a monoclonal antibody named as 10-7G mAb was characterized in further experiments. However, Western blot analysis showed the 10-7G mAb predominantly recognized the Hpt α chain whereas polyclonal Hpt Ab recognized the Hpt β chain as well as Hpt α chain (Figure 1C). Very faint band pf pro-haptoglobin (proHpt) was observed in Western blot, using 10-7G mAb. These results indicated that the 10-7G mAb recognized the Hpt α chain under reducing condition.

### Characterization of 10-7G mAb by column chromatography followed by Western blot and mass spectrometry analyses

**Figure 2:**
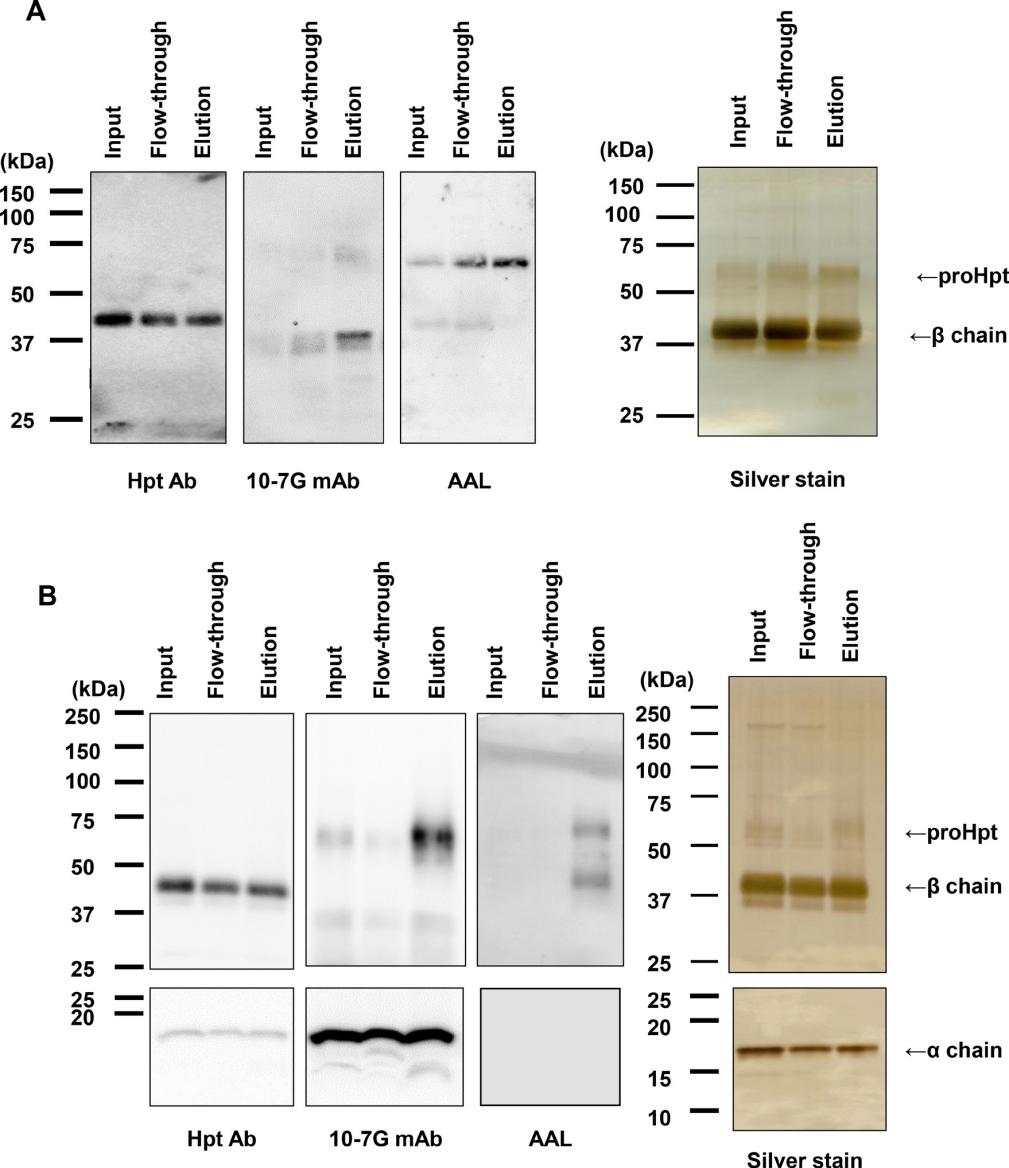
Affinity chromatography with the 10-7G mAb followed by Western blot analyses Western blot analyses with Hpt Ab and the 10-7G mAb and AAL blot analysis were performed for three fractions under affinity chromatography with the 10-7G mAb. Hpt products (**A**) and a sample consisting of a mixture of Hpt products and 1% fHpt-HCT purified from Hpt-transfected HCT116 cells (**B**) were used in chromatography. To detect Hpt α chain with Western blot, 15% SDS-PAGE was used. Silver staining was shown as loading control. Detail procedure was described in Materials and Methods.

**Figure 3:**
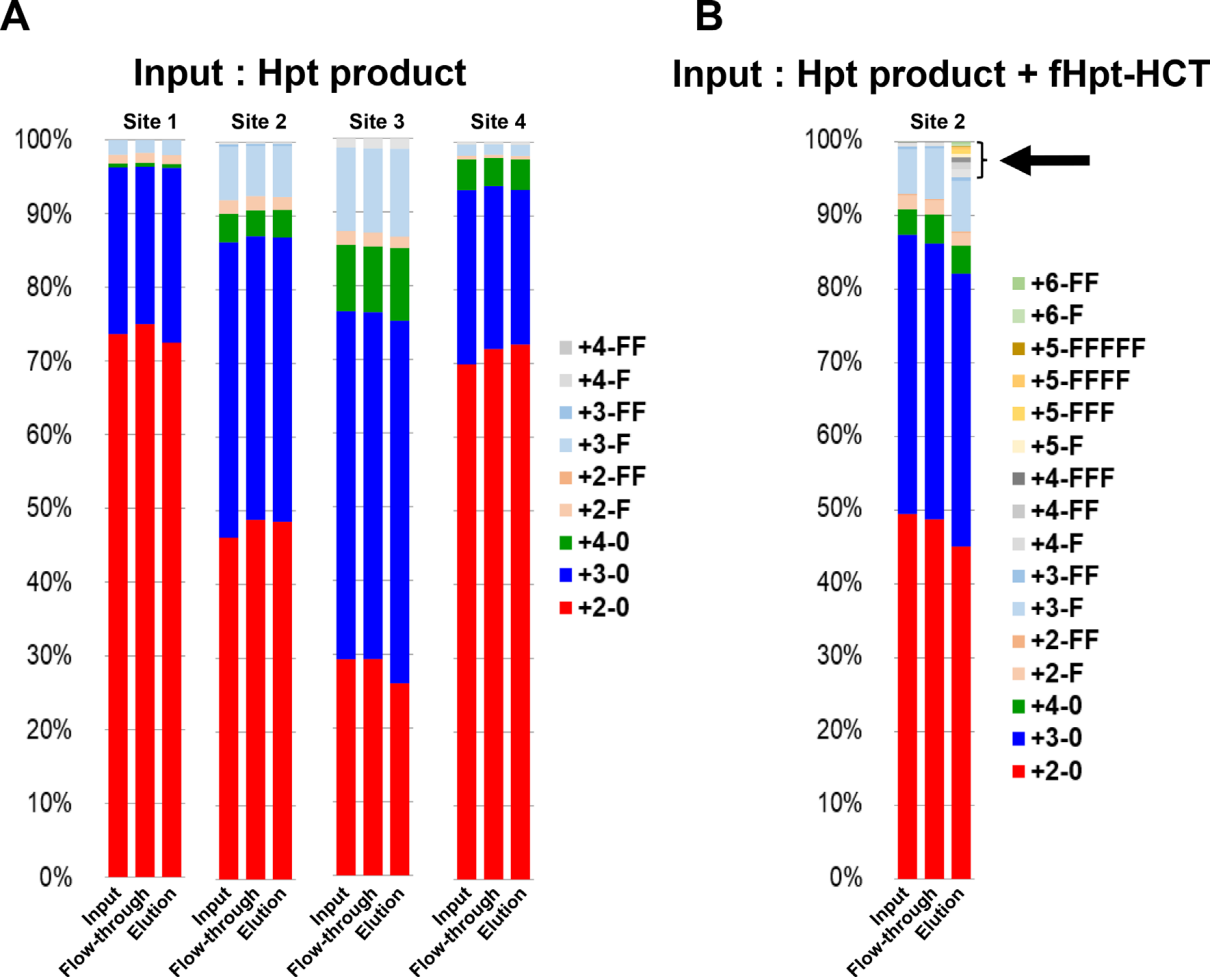
Affinity chromatography with the 10-7G mAb followed by site-specific analyses of *N*-glycans on haptoglobin by mass spectrometry The ratio of various *N*-glycans at each site on haptoglobin was calculated from the results of mass spectrometry for three fractions (input, flow-through, elution) under affinity chromatography with the 10-7G mAb. Hpt products (**A**) and a sample consisting of a mixture of Hpt products + 1% fHpt-HCT purified from Hpt-transfected HCT116 cells (**B**) were used in chromatography. The arrow indicates highly branched *N*-glycans on Fuc-Hpt and/or proHpt derived from HCT116 cells. Site 1: Asn 184; Site 2: Asn 207; Site 3: Asn 211; Site 4: Asn 241. For the abbreviations of *N*-glycan structures on the glycopeptides, the simplified notation was used. For example, the peptide containing the tri-antennary *N*-glycan with one Fuc residue is represented as “3-F.” The first numeral indicates the branch number (tri-antennary in this case) or LAcNAc number (3 LAcNAc in this case), and “F” indicates one Fuc residue. “0” denotes the absence of Fuc.

The 10-7G mAb was not reactive to Hpt derived from a healthy volunteer in ELISA (Figure 1B), although the 10-7G mAb recognized Hpt α chain in Western blot (Figure 1C). To examine the reactivity of the 10-7G mAb in native conditions, Hpt product was applied to an affinity column immobilized with the 10-7G mAb. The results of Western blot analyses with Hpt Ab (Dako) showed equal amounts of mature Hpt in three fractions (input, flow-through, and elution; left panel in Figure 2A), indicating that the 10-7G mAb did not concentrate mature Hpt before and after affinity chromatography. ProHpt detected by the 10-7G mAb was enriched by affinity chromatography (center panel in Figure 2A) and proHpt was strongly fucosylated as judged by AAL lectin blot (right panel in Figure 2A). The enrichment of proHpt was verified more clearly when a small amount of fHpt-HCT was added to Hpt product (center panel in Figure 2B). Additionally, Fuc-Hpt was concentrated by affinity chromatography as shown in AAL blot analysis (right panel in Figure 2B). These results indicate that the 10-7G mAb recognizes Fuc-Hpt as well as proHpt in native conditions. To verify the enrichment of Fuc-Hpt and/or proHpt by 10-7G-affinity chromatography in detail, site-specific structural analysis of *N*-glycans on Hpt was performed (Figure 3). Consistent with our previous report, a small amount of di-, tri-, and tetra-antennary fucosylated glycans were mainly observed at site 3 (Asn 211) and to a lesser extent at site 2 (Asn 184) of Hpt product. No change was observed in the glycan profile of Hpt product with mass spectrometry analyses before and after affinity chromatography (Figure 3A), although proHpt detected by AAL blot analysis was condensed (Figure 2A). However, when a small amount of fHpt-HCT was added to Hpt product, highly branched fucosylated glycans derived from Fuc-Hpt and/or proHpt contained in fHpt-HCT were enriched in the elution fraction following affinity chromatography (Figure 3B), consistent with the results obtained from the AAL blot analysis shown in Figure 2B. These results indicate that the 10-7G mAb predominantly recognizes proHpt and /or Fuc-Hpt in native conditions.

### Prohaptoglobin is secreted by several kinds of cancer cell lines

**Figure 4:**
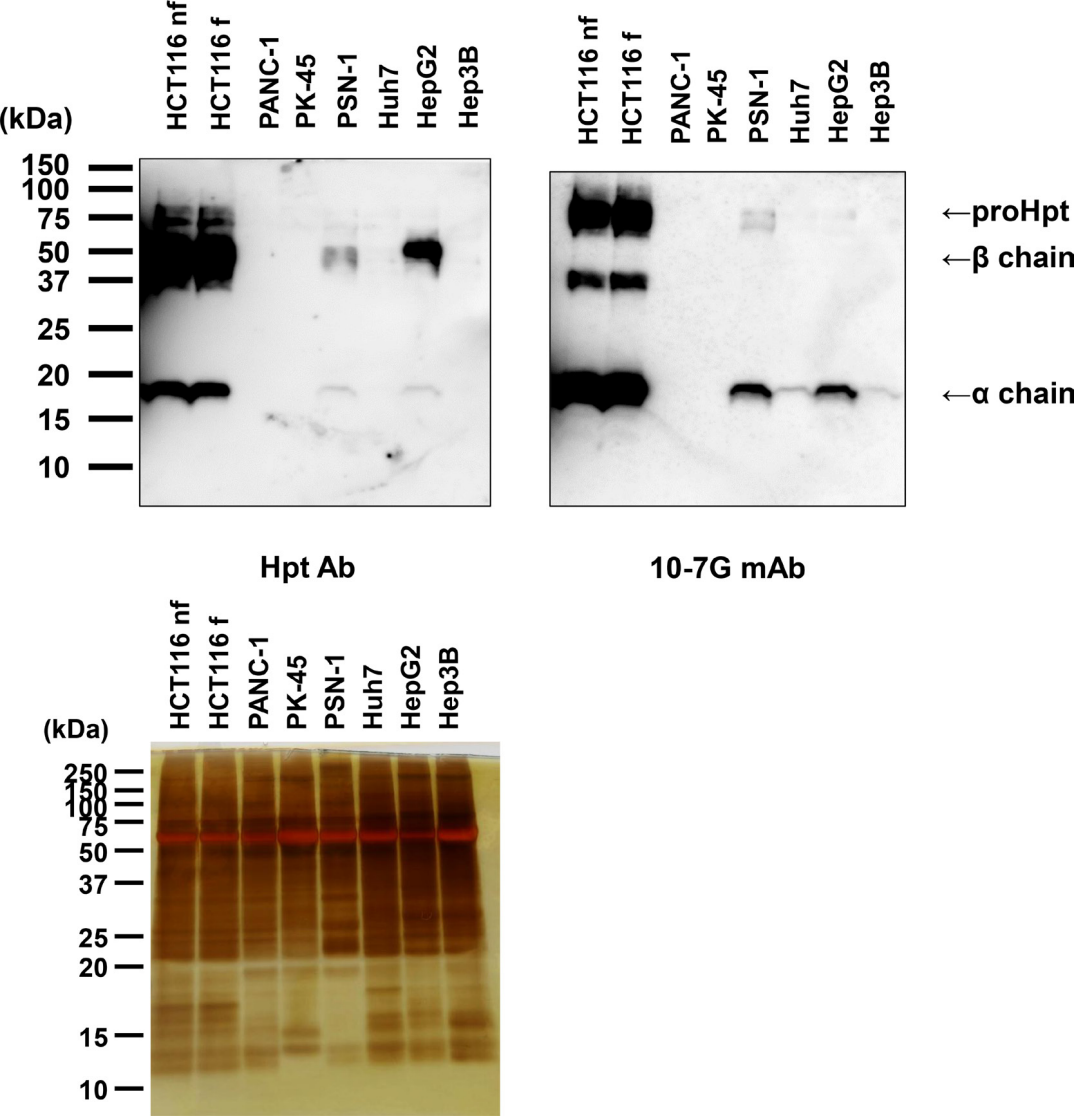
Western blot analysis of proHpt with the 10-7G mAb in conditioned media from human pancreatic cancer and hepatocellular carcinoma cell lines Thirty micrograms of secreted proteins in conditioned media were electrophoresed on 15% SDS-PAGE gels, followed by Western blot analyses with Hpt Ab and the 10-7G mAb. Samples collected from HCT116 cells with or without L-fucose treatment (HCT116 f and HCT116 nf, respectively) were used as positive controls. Each band showed Hpt-related protein as shown at right side. Silver staining was shown as loading controls.

As shown in Supplementary Figure 1, proHpt was increased in the sera of patients with pancreatic cancer. To investigate which kinds of cell types produce proHpt, Western blot analyses under reducing conditions by the Hpt Ab (Dako) and the 10-7G mAb were performed using several kinds of pancreatic cancer and hepatocellular carcinoma cell lines. As shown in Figure 4, the human pancreatic cancer cell line PSN-1 secreted mature Hpt (left panel in Figure 4). PSN-1 also secreted a substantial amount of proHpt (right panel in Figure 4). Two other pancreatic cancer cell lines, PANC-1 and PK-45, did not produce both mature Hpt and proHpt. All three hepatocellular carcinoma cell lines tested, Huh7, HepG2, and Hep3B secreted mature Hpt, although Hpt levels in Huh7 and Hep3B cells were smaller than that of HepG2 cells. In contrast, only HepG2 cells secreted small amount of proHpt. Transfectants of Hpt in HCT116 cells produced large amounts of Hpt including proHpt. Western blot using 10-7G mAb showed strong binding to Hpt α chain band. These results suggest that certain kinds of pancreatic cancer and hepatocellular carcinoma cell lines could produce proHpt.

### Hepatocytes surrounding metastasized pancreatic cancer cells produce the 10-7G mAb-reacting Hpt

**Figure 5:**
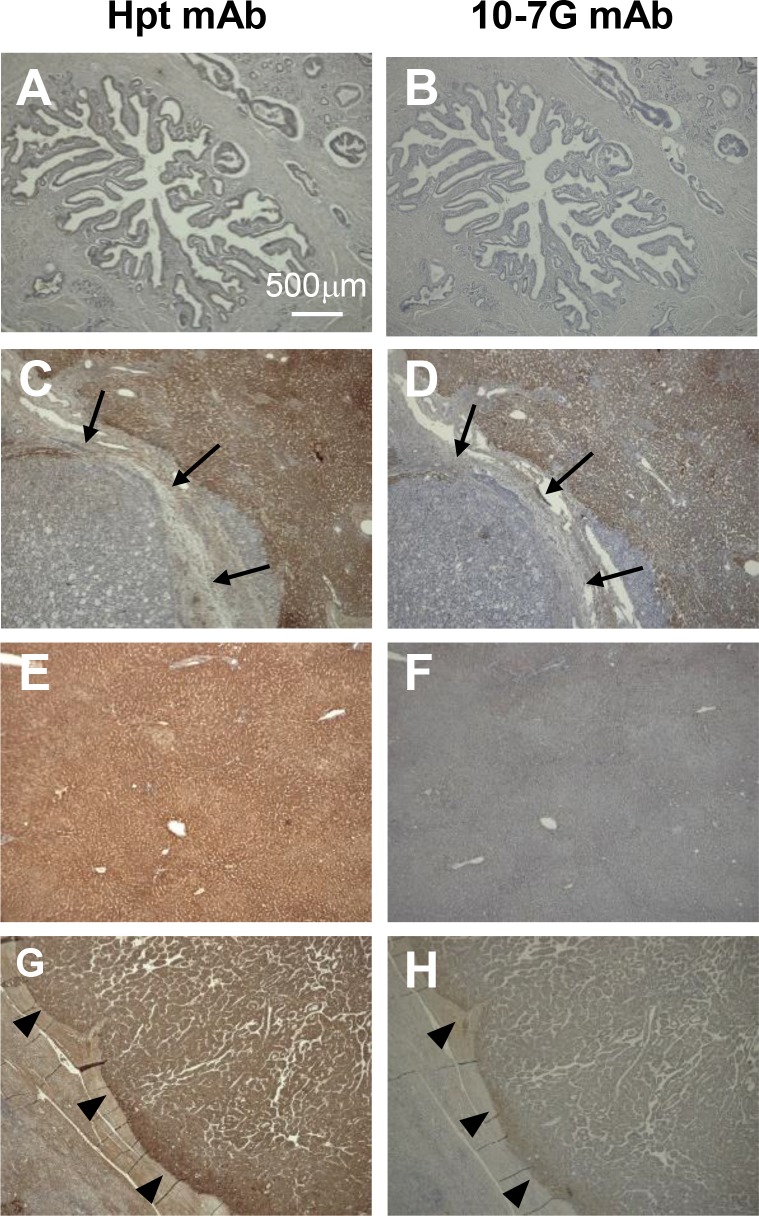
Immunohistochemical study of Fuc-Hpt in pancreatic cancer and liver tissue Immunohistochemical staining was performed with both the Hpt mAb and the 10-7G mAb as described in the Material and Methods section. (**A**) and (**B**), primary pancreatic cancer; (**C**) and (**D**), metastatic pancreatic cancer to the liver; (**E**) and (**F**), normal hepatic tissue; (**G**) and (**H**), hepatocellular carcinoma (HCC). Photographs were taken using a 4× objective. *Scale bar,* 500 μm. Arrow showed metastatic pancreatic cancer in the liver and arrowhead showed positive staining of each antibody.

To identify which kinds of cell types mainly produce Fuc-Hpt and/or proHpt in pancreatic cancer patients, we performed immunohistochemical analysis with the 10-7G mAb using various tissues. As shown in Figure 5, weak expression of mature Hpt was detected by the monoclonal anti-Hpt antibody (Abcam) in both primary and metastasized pancreatic cancer cells (Figures 5A and 5C). Hepatocytes surrounding metastatic pancreatic cancer tissue and normal hepatic tissue strongly expressed mature Hpt (Figure 5C and 5E). In contrast, neither primary nor metastasized pancreatic cancer cells were stained by the 10-7G mAb (Figure 5B and 5D). Strikingly, hepatic tissues surrounding the metastatic pancreatic cancer tissue were stained by the 10-7G mAb, whereas normal hepatocytes were not stained (Figure 5D and 5F). These results suggest that pancreatic cancer cells that have metastasized to the liver promote the production of Fuc-Hpt and/or proHpt (cancer associated Hpt) from adjacent hepatocytes. In the case of hepatocellular carcinoma, tumor cells were stained with both Hpt Ab and the 10-7G mAb (Figure 5G and 5H), indicating that hepatoma cells produce both mature Hpt and Fuc-Hpt. In a case of liver metastasis of colorectal cancer, strong staining with the 10-7G mAb was observed in adjacent lesion of the metastasis and the staining level was decreased in distant lesion of the metastasis (Supplementary Figure 2).

### Serum Fuc-Hpt levels determined with our newly developed ELISA were increased in patients with pancreatic cancer and colon cancer

**Figure 6:**
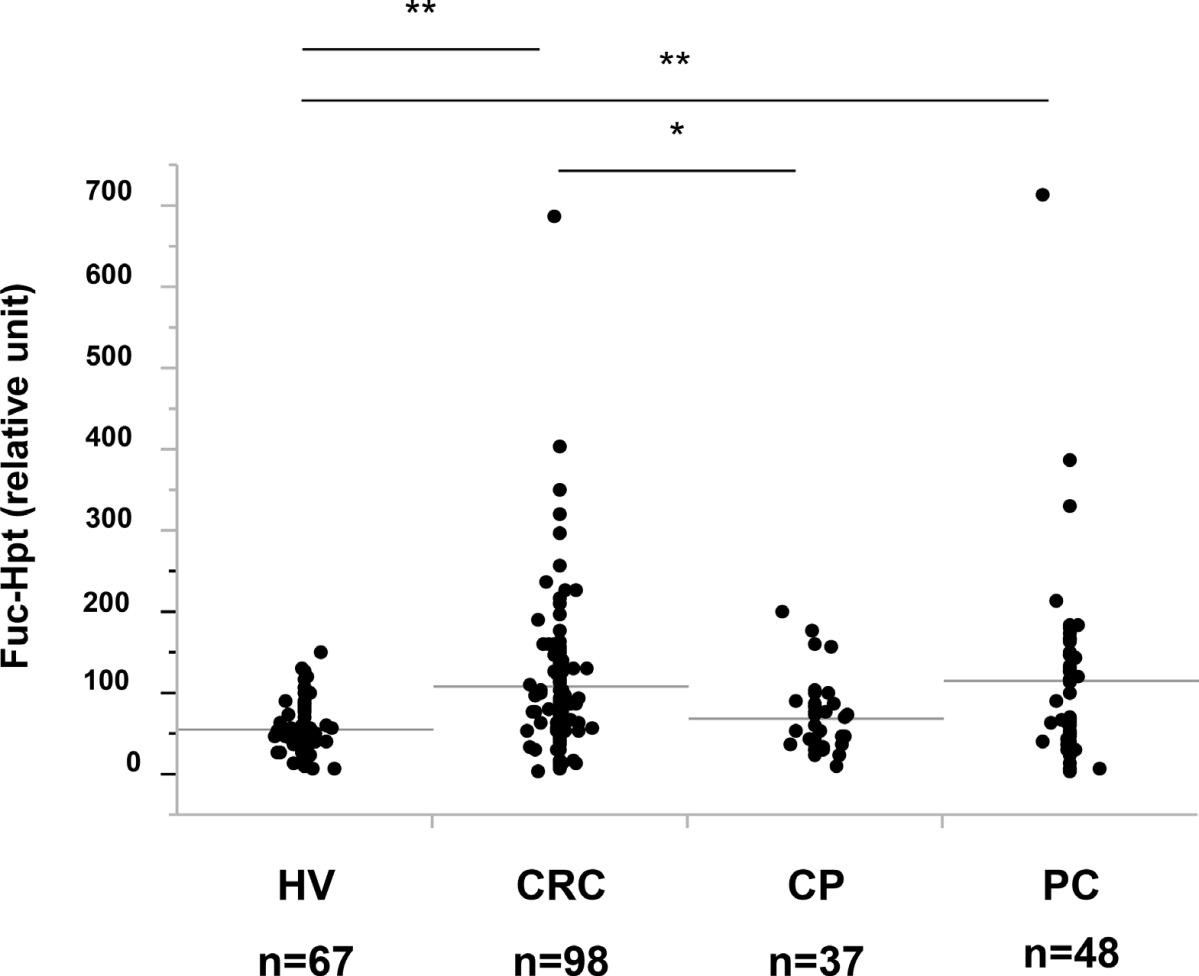
Determination of serum Fuc-Hpt levels with new ELISA system, using 10-7G mAb Serum Fuc-Hpt levels in healthy volunteers (HV), colorectal cancer patients (CRC), chronic pancreatitis patients (CP) and pancreatic cancer patients (PC), were measured with new ELISA system with 10-7G mAb. ^*^P < 0.05. ^**^P < 0.01.

**Figure 7:**
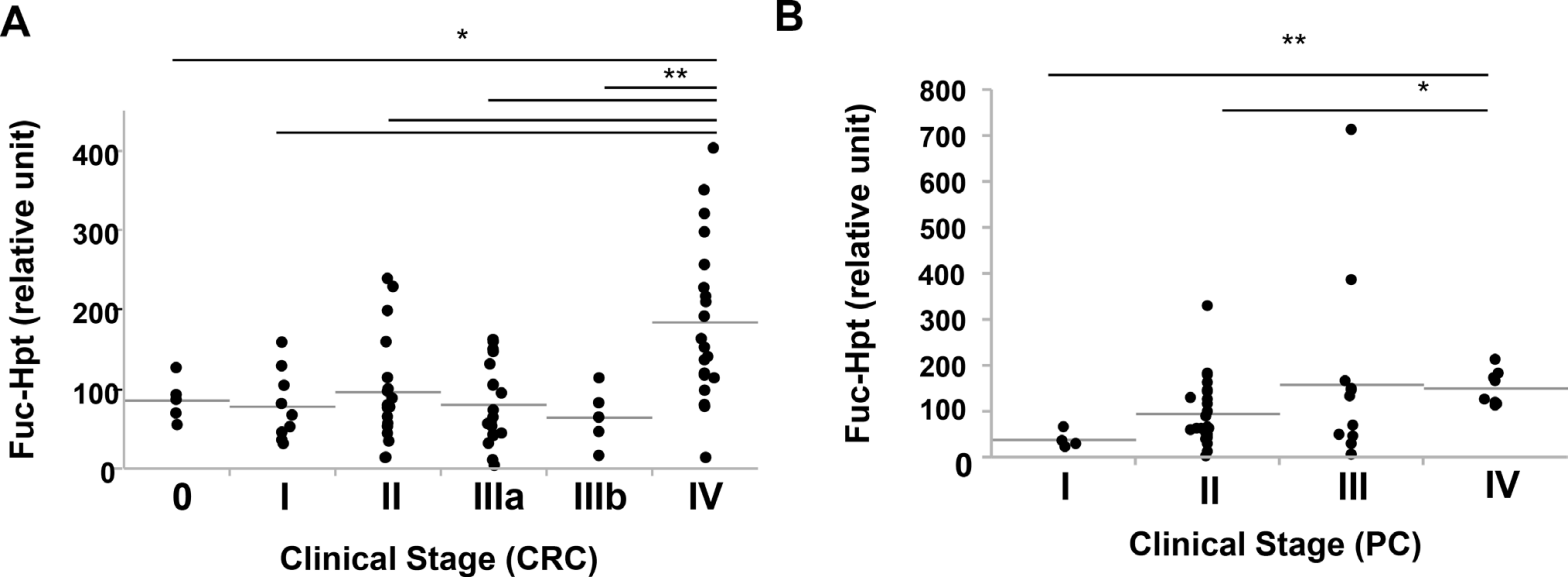
Relationship of clinical stage and serum Fuc-Hpt levels determined with new ELISA system, using 10-7G mAb Serum Fuc-Hpt levels were plotted in terms of clinical stage of colorectal cancer (CRC) (**A**) and pancreatic cancer (PC) (**B**). Horizontal grey lines indicate the mean values of Fuc-Hpt in each group. ^*^P < 0.05. ^**^P < 0.01.

We previously reported that serum Fuc-Hpt levels determined with lectin-antibody ELSA were a diagnosis biomarker for pancreatic cancer [5] and a prognostic biomarker for colorectal cancer [9]. Therefore, we measured serum Fuc-Hpt levels of those patients with our newly developed ELISA. First, we compared Fuc-Hpt levels determined with 2 different ELISA methods. As shown in Supplementary Figure 3, The correlation between 2 kinds of methods was not statistically significant, but tended to be slightly similar. Interestingly serum Fuc-Hpt levels determined with our newly developed ELISA were significantly increased in patients with pancreatic and colorectal cancer patients (Figure 6). A few cases of chronic pancreatitis showed high levels of Fuc-Hpt determined with newly developed ELISA. In cases of colorectal cancer, the levels were significantly increased at clinical stage IV, especially in liver metastasis (Figure 7A). In contrast, significant increases in Fuc-Hpt levels determined with newly developed ELISA were observed in clinical stage III and IV of pancreatic cancer (Figure 7B).

## DISCUSSION

In the present study, we have developed antibodies specific for Fuc-Hpt using a unique screening system with GMDS mutant HCT116 cells and characterized one of the established antibodies, named the 10-7G mAb. We demonstrated that the 10-7G mAb predominantly recognized Hpt α chain as well as proHpt in the reducing conditions and that this antibody recognized both Fuc-Hpt and proHpt in native conditions. Additionally, we found that the serum level of a novel Fuc-Hpt determined by 10-7G mAb was increased in patients with pancreatic and colorectal cancer at the advanced stage and that expression of Fuc-Hpt was not observed in primary or metastasized pancreatic cancer cells but observed in hepatocytes surrounding metastatic pancreatic cancer.

Mature human Hpt, which is produced mainly by the liver and functions primarily to bind free plasma hemoglobin released from erythrocytes due to intravascular hemolysis, consists of an α and β chain heterodimer combined by disulfide bonds as a basic unit which eventually dimerizes or multimerizes [15–18]. The β chain possesses all of the four *N*-glycans in mature Hpt at the sites of Asn 184, 207, 211, and 241, where fucose residues are additionally bound by fucosyltransferases in cancer patients [10, 19–21]. ProHpt is a premature form of haptoglobin containing the α and β subunits within a single polypeptide chain; proHpt is separated and joined again via disulfide bonds in the maturation process. The separation at the junction of the α and β chains is catalyzed by a serine protease named complement C1r-like proteinase (C1r-LP) in the endoplasmic reticulum, before the protein enters the Golgi complex [22–25].

The 10-7G mAb showed a positive reaction to PC-Hpt and a negative reaction to HV-Hpt (Figure 1B). This is partially due to the relatively higher level of proHpt in PC-Hpt than in HV-Hpt (Supplementary Figure 1). Since C1r-LP activity is primarily high in the liver, proHpt is scarcely produced in hepatocytes under physiological conditions. In fact, normal liver tissue was not stained with the 10-7G mAb recognizing proHpt (Figure 5F). It has been reported that various tissues including the lung, arteries, kidneys, skin, and leukocytes express extra-hepatic Hpt [26–30]. Therefore, proHpt, which is detected in the sera of healthy volunteers, might be produced by non-hepatic tissues/cells, where both Hpt and C1r-LP expression is relatively low. On the other hand, the 10-7G mAb positively stained hepatic tissue surrounding metastatic pancreatic cancer (Figure 5D) as well as primary hepatoma cells (Figure 5H), where mature Hpt is primarily produced. However, we could not demonstrate that this staining is dependent on Fuc-Hpt and/or proHpt. This is the limitation of immunostaining of 10-7G mAb.

The binding epitope of proHpt for the reactivity of the 10-7G mAb still remains to be identified. The results of present experiments indicate that haptoglobin α chain, which does not possess *N*-glycans, is a critical part for the binding of the 10-7G mAb which recognizes Fuc-Hpt and proHpt. While the detailed molecular mechanisms remain unknown, conformational changes following fucosylation and/or N-glycan branching may affect the binding between α chain and the 10-7G mAb.

Recently, various functions of mature Hpt other than its main role of binding free hemoglobin in the circulation have been described, including immune modulation [31–33], angiogenesis [34], arterial restructuring [35], cell proliferation and cell migration [36]. In contrast, limited information about the function of proHpt is available. Oh *et al.* recently reported that proHpt plays a pivotal role in angiogenesis through the upregulation of vascular endothelial growth factor (VEGF) and VEGF receptor 2 expression in endothelial cells [37]. The present study demonstrated increased proHpt levels in the sera of patients with pancreatic cancer (Figure 6 and Supplementary Figure 1), consistent with the study of Oh *et al.* reporting an increase in serum proHpt levels in patients with hepatoma, suggesting a role for proHpt in cancer progression [37]. Therefore, proHpt could be considered as cancer-associated Hpt.

Our preliminary ELISA kit measuring Fuc-Hpt showed relatively similar results of our previous reports by lectin-antibody ELISA while there is slight correlation of 2 types of Fuc-Hpt measured by different methods (Supplementary Figure 2). Basically, serum Fuc-Hpt level determined with 10-7G mAb is increased at the advanced stage, especially liver metastasis. In certain cases of pancreatic cancer, Fuc-Hpt levels were increased at clinical stage II and III. However, micro-metastasis at different organs, which were not detected with image diagnosis such as CT or MRI would be observed at clinical stage II and III of pancreatic cancer. Therefore, follow-up studies on serum Fuc-Hpt levels should be performed before/after surgical treatment. Since antibody is much superior to lectin in terms of specificity and reproducibility in general, our newly developed Fuc-Hpt ELISA could be applied to clinical diagnosis test for detecting early liver metastasis. However, we could not demonstrate that serum level of Fuc-Hpt using a novel ELISA kit is dependent on Fuc-Hpt and/or proHpt. This is the limitation of ELISA of 10-7G mAb.

In conclusion, we succeeded in the establishment of a monoclonal antibody recognizing Fuc-Hpt and proHpt and investigated the clinical availability with immunohistochemistry and ELISA. The 10-7G mAb might be a promising tool to predict liver metastasis of pancreatic cancer or colorectal cancer in a serum-based test.

## MATERIALS AND METHODS

### Clinical samples

All clinical samples were obtained from Osaka University affiliated hospitals or Osaka Medical Center for Cancer and Cardiovascular Diseases. Sera from healthy volunteers who received physical checkups or patients with pancreatic cancer, chronic pancreatitis and colorectal cancer before medical interventions were collected and kept frozen at –80°C until use. Tissue samples obtained from patients with primary or metastatic pancreatic cancer and hepatocellular carcinoma at primary surgery were embedded in paraffin sections and kept at room temperature until use. This study was approved by the ethics committee of Osaka University Hospital (No. 11300-3), and was conducted in accordance with approved guidelines. Written informed consent was obtained from all subjects in this study at the time of enrollment or blood sampling.

### Haptoglobin products

Hpt product purified from pooled plasma of several healthy individuals was purchased from Japan Blood Products Organization (Tokyo, Japan).

### Cell culture

The human colon cancer cell line HCT116; human pancreatic cancer cell lines PANC-1, PK-45, and PSN-1; and human hepatocellular carcinoma cell lines Huh7, HepG2, and Hep3B were obtained from the American Type Culture Collection (ATCC, Manassas, VA) or the Cell Resource Center for Biomedical Research, Institute of Development, Aging and Cancer, Tohoku University (Sendai, Japan). They were maintained at 37°C in a humidified atmosphere with 5% CO_2_ and 95% air in RPMI 1640 (Sigma-Aldrich Corp., St. Louis, MO), supplemented with 10% heat-inactivated fetal bovine serum (Life Technologies Corp., Carlsbad, CA), 100 units/ml of penicillin and 100 μg/ml of streptomycin (Nacalai Tesque Inc., Kyoto, Japan).

### Purification of haptoglobin from conditioned media from HCT116 cells, or from human sera

Because HCT116 cells have a mutation in the fucosylation regulatory gene encoding GDP-mannose-4,6-dehydratase (*GMDS*) [14], all glycoproteins produced from HCT116 cells lack fucosylation. In contrast, fucosylation is restored with L-fucose treatment. An expression vector (pcDNA/hygro) with human Hpt encoding Hpt α2β (Hpt2) [38] was transfected into HCT116 cells. Cells expressing high levels of Hpt were cloned following treatment with Hygromycin B (Wako Pure Chemical Industries Ltd., Osaka, Japan). Non-fucosylated Hpt (Non-fHpt-HCT) was purified from conditioned media from Hpt-transfected HCT116 cells using an affinity chromatography column coupled with a rabbit polyclonal anti-Hpt antibody (Hpt Ab, Dako, Glostrup, Denmark) as previously described [3]. Fucosylated Hpt (fHpt-HCT) was purified by the same procedure after the addition of 1 mM L-fucose (Wako) into the media from HCT116 cells. Hpt purified from the serum of a healthy volunteer (HV-Hpt) or from a patient with pancreatic cancer (PC-Hpt) was obtained using affinity chromatography as well. In the affinity chromatography, PBS was used for washing columns and diluting samples.

### Hybridoma production and the establishment of monoclonal antibodies against Fuc-Hpt

Balb/c mice were immunized at 6 weeks of age by an intramuscular injection of 200 μg of purified fHpt-HCT. Additional immunizations of 50 μg of purified fHpt-HCT were administered twice every two weeks, and the splenocytes were extracted following confirmation of a rise in antibody titer against Hpt. All experimental protocols and animal maintenance procedures used in this study were approved by the Ethics Review Committee for Animal Experimentation of Osaka University Graduate School of Medicine (IACUC 25038). The splenocytes were fused with mouse myeloma cells to make hybridomas according to a standard procedure. Conditioned media from growing hybridomas were screened using ELISA, as described below. Selection was performed based on a positive reaction to fHpt-HCT and a negative reaction to Non-fHpt-HCT in the first screening, and a positive reaction to PC-Hpt and a negative reaction to HV-Hpt in the second screening.

### Coomassie Brilliant Blue (CBB) staining, silver staining, Western blot, and lectin blot analysis

Equal amounts of purified proteins or secreted proteins in conditioned media from each cancer cell line, or equal aliquots of sera were denatured in SDS sample buffer with 2-mercaptoethanol by boiling at 95°C, resolved on 10% SDS-PAGE gels and separated by electrophoresis. 15% SDS-PAGE was used to detect Hpt α-chain with Western blot. For CBB staining or silver staining, gels were stained and destained according to a standard procedure. For Western blot or lectin blot analysis, the proteins were transferred onto PVDF membranes and the membranes were blocked with 5% skim milk in Tris-buffered saline containing 0.05% Tween 20 (TBS-T) or 3% BSA in TBS-T. The membranes were incubated with Hpt Ab (Dako), the established mouse monoclonal 10-7G antibody (10-7G mAb), or biotinylated AAL (J-OIL MILLS Inc., Tokyo, Japan) and then incubated with HRP-conjugated secondary antibody (Promega Corp., Madison, WI) or streptavidin-HRP (Abcam plc., Cambridge, UK) as previously described [3]. After each incubation step, the membranes were washed with TBS-T three times for 10 min each. Protein bands were visualized by developing the blots with Chemiluminescent Reagent ImmunoStar Zeta (Wako, Osaka, Japan) using ImageQuant LAS 4000mini (GE Healthcare, Buckinghamshire, UK). The cleavage of *N*-glycans in purified Hpt was performed using glycopeptidase F (Peptide *N*-glycosidase F, Takara Bio Inc., Shiga, Japan) according to the manufacturer's protocol. As a negative control, an equal amount of distilled water was added to the samples instead of the enzyme. To analyze smaller proteins such as haptoglobin α chain, 15% SDS-PAGE gels were used.

### Site-specific analyses of N-glycans on haptoglobin by mass spectrometry

The detailed procedure is as previously described [19]. Briefly, 50 μg of Hpt was digested with trypsin and endoprotease Glu-C after reduction (dithiothreitol) and alkylation (iodoacetamide). Glycopeptides were enriched using a Sepharose CL4B column. For de-sialylation, the glycopeptides were dissolved in 2 M acetic acid and incubated for 2 h at 80°C. Dried glycopeptides were dissolved in 12 μL of 0.08% formic acid and were subjected to liquid chromatography electrospray ionization (LC-ESI) MS analyses. Analytical conditions for LC-ESI MS and the data analysis procedure for relative quantitation have been previously described [39]. Briefly, glycopeptides were separated using an octadecyl silica (ODS) column under specific gradient conditions at a flow rate of 50 μL/min using an Accela HPLC system (Thermo Fisher Scientific Inc., Waltham, MA). The mobile phases were solvent A (0.08% formic acid) and solvent B (0.15% formic acid in 80% acetonitrile). The elution fraction was introduced continuously into an ESI source, and glycopeptides were analyzed by LTQ Orbitrap XL (Thermo) in positive ion mode. For the relative quantitation of site-specific *N*-glycans, the peak intensity of quadruply charged ions [M+4H]^4+^ for site 1 (Asn 184) or 4 (Asn 241) and triply charged ions [M+3H]^3+^ for site 2 (Asn 207) or site 3 (Asn 211) on an average mass spectrum were used (Xcalibur software ver. 2.0.7; Thermo).

### Immunohistochemistry

Tissue samples fixed in 10% formaldehyde and embedded in paraffin sections (4 μm thick) were de-paraffinized in xylene and rehydrated in graded ethanol. Endogenous peroxidase activity was blocked by incubating 3% hydrogen peroxide in 100% methanol. After blocking non-specific protein binding with Protein Block Serum-Free (Code X0909, Dako) for 10 min at room temperature (RT), the sections were incubated with 3.3 μg/mL of a mouse monoclonal anti-Hpt antibody (Abcam) or 5 μg/mL of the 10-7G mAb diluted in Antibody Diluent (Code S0809, Dako) overnight at 4°C. The sections were then incubated with EnVision+ System HRP-Labeled Polymer Anti-Rabbit (Code K4002, Dako), or Anti-Mouse (Code K4000, Dako) for 30 min at RT. After each incubation step, the sections were washed with PBS three times for 10 min each. Subsequently, the sections were incubated with the Liquid DAB+ Substrate Chromogen System (Code 3467, Dako) for 5 min to develop the stain. Finally, the sections were counterstained with haematoxylin, dehydrated, and mounted. Pictures were taken with a BZ-9000 microscope (KEYENCE, Tokyo, Japan).

### ELISA

For hybridoma screening, conditioned media from mouse hybridomas or control fluids were added on Nunc Maxisorp 96-well immunoplates (Thermo) pre-coated with a rabbit anti-mouse IgG antibody and incubated for 1 h at RT. Purified Hpt were loaded on the plate at 100 ng/mL for 1 h at RT followed by solid-phase blocking. HRP-conjugated Hpt Ab (diluted 1:2,000) was added to the plate and incubated for 1 h at RT. After each incubation step, the plate was washed with PBS three times. The color was developed using an o-phenylenediamine dihydrochloride tablet as the chromogen, and the optical density was measured at 492 nm by an iMark^TM^ Microplate Absorbance Reader (Bio-Rad Laboratories Inc. Philadelphia, PA). For development of a new ELISA kit to measure Fuc-Hpt, the 10-7G mAb was coated on 96-well plate. After blocking with TBS containing 10% Block Ace (DS Pharma Biomedical, Osaka, Japan) 5000 times diluted serum samples were loaded. The plate was incubated for 1 h at RT and then washed with TBS containing 0.05% Tween 20. HRP-conjugated the 3-25-1C mAb, which is another established antibody specific for Fuc-Hpt was added to each well and incubated for 1 h at RT. The development procedure was the same as described above.

### Statistical analysis

The statistical analysis was performed using JMP Pro 12.0 software (SAS Institute Inc., Cary, NC). The statistical analysis included descriptive statistics, analysis of variance, the Wilcoxon and Kruskal-Wallis tests, and Spearman R correlation analysis. As the measurement data from 10-7G mAb ELISA and lectin-antibody ELISA did not show Gaussian distribution; these parameters were common log-transformed before correlation analysis. Differences were considered to be statistically significant at a *P*-value < 0.05.

## SUPPLEMENTARY MATERIALS FIGURES



## Abbreviations

AAL: *Aleuria aurantia lectin*
ELISA: enzyme-linked immunosorbent assay
Hpt: haptoglobin
Fuc-Hpt: fucosylated haptoglobin
CA-Hpt: Cancer-associated haptoglobin
fHpt-HCT: fucosylated haptoglobin produced by HCT116 cells with L-fucose supplementation
Non-fHpt-HCT: non-fucosylated haptoglobin produced by HCT116 cells without L-fucose supplementation
ProHpt: prohaptoglobin
CRC: Colorectal cancer
PC: Pancreatic cancer
CP: Chronic pancreatitis
HV: Healthy volunteer
mAb: monoclonal antibody
GMDS: GDP-mannose-4,6-dehydratase
