# Supplementary material for: Establishment of an antibody specific for cancer-associated haptoglobin: a possible implication of clinical investigation
Source: Oncotarget. 2018 Jan 29;9(16):12732–44. doi: 10.18632/oncotarget.24332 (PMC5849169; doi:10.18632/oncotarget.24332)
Supplement: Supplementary file 1 [file oncotarget-09-12732-s001.pdf]

Establishment of an antibody specific for cancer-associated haptoglobin: a possible implication of clinical investigation

SUPPLEMENTARY MATERIALS

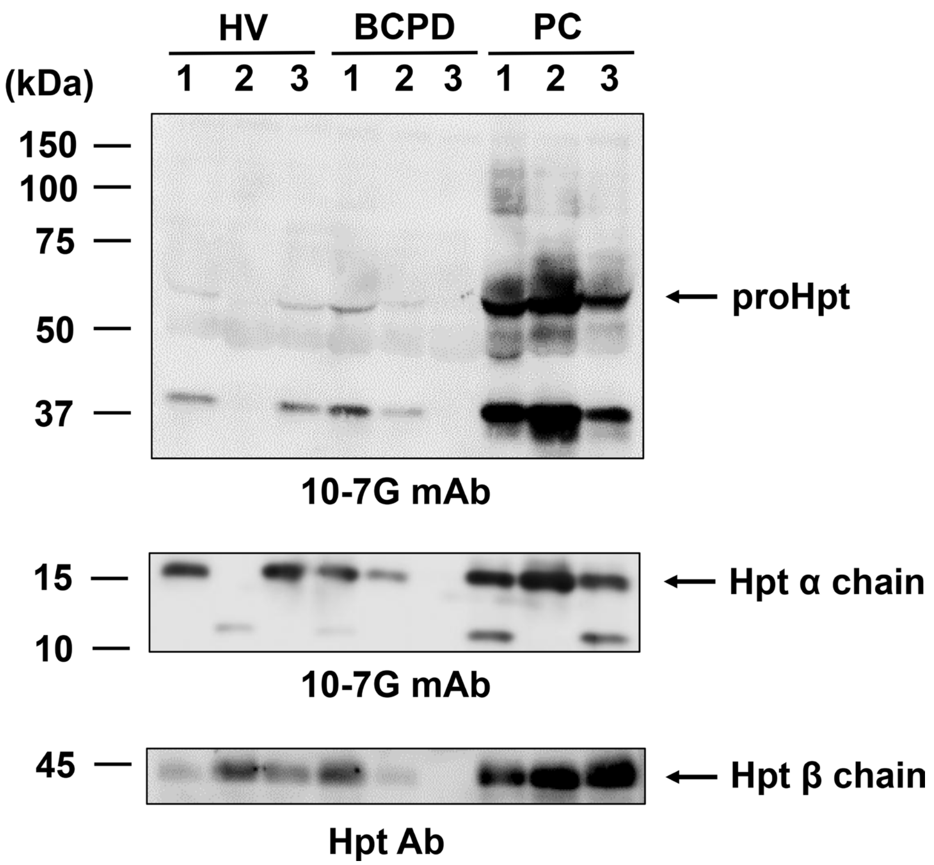

Supplementary Figure 1: Western blot analysis of proHpt with the 10-7G mAb, using human serum samples. A 0.5 µl volume of sera from healthy volunteers (HV, $n = 3$), patients with benign cholangio-pancreatic diseases (BCPD, $n = 3$) and pancreatic cancer (PC, $n = 3$) were electrophoresed on 10% SDS-PAGE gels followed by Western blot analyses with the 10-7G mAb and the Hpt Ab.

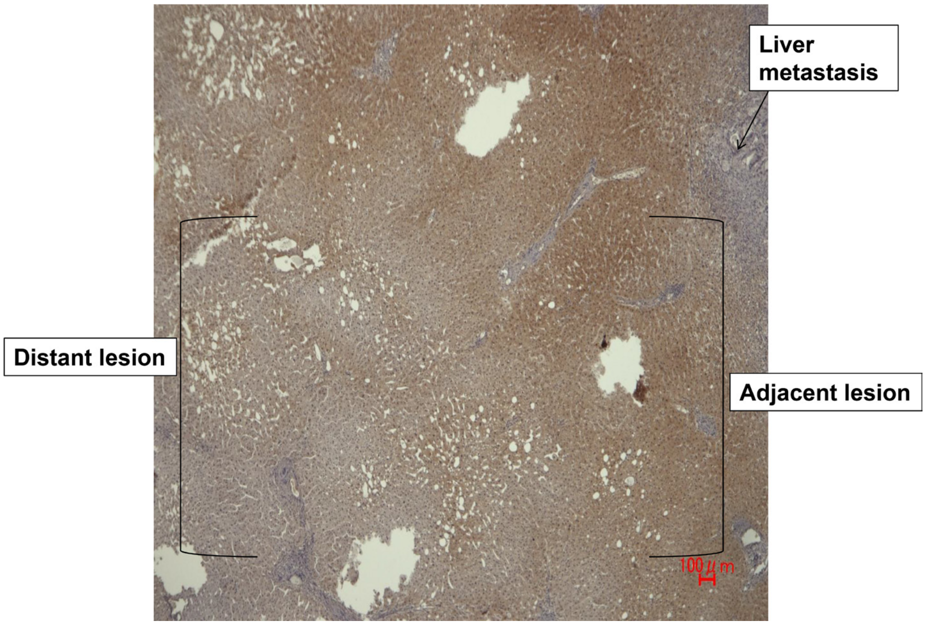

Supplementary Figure 2: Immunohistochemical staining of Fuc-Hpt with 10-7G mAb. Positive staining was observed in the surrounding hepatocytes of liver metastasis of colorectal cancer. The staining levels were higher in surrounding lesion of the metastasis than in distant lesion of the metastasis.

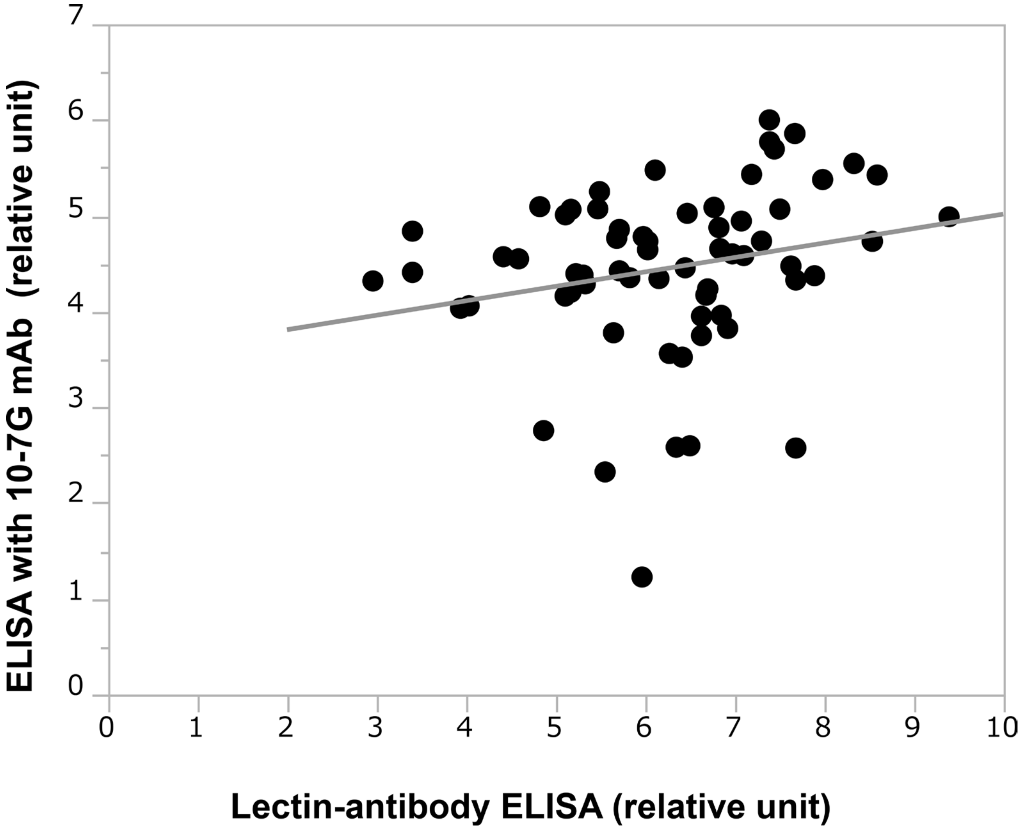

Supplementary Figure 3: Correlation of serum Fuc-Hpt and CA-Hpt levels determined by 2 different methods. Serum Fuc-Hpt levels were measured with conventional lectin-antibody ELISA and new ELISA system with 10-7G mAb. Each data was described by Log value. Sera of 64 colorectal cancer patients were used in this analysis. The correlation between 2 kinds of methods was not statistically significant, but tended to be slightly similar ($R^2 = 0.05$, $P = 0.076$).
